# Green Synthesis and Application of Biochar Derived from Alien Vegetation Wood for Proton Exchange Membrane Fuel Cells

**DOI:** 10.1002/open.202500025

**Published:** 2025-04-26

**Authors:** Alunge Gift Sobekwa, Nakedi Albert Mojapelo, Evan David Visser, Ntalane Sello Seroka, Lindiwe Khotseng

**Affiliations:** ^1^ Department of Chemistry Faculty of Natural Sciences University of the Western Cape Robert Sobukwe Road, Private Bag X17 Bellville 7535 South Africa; ^2^ Energy Centre Smart Places Cluster Council for Science and Industrial Research (CSIR) Pretoria 0001 South Africa

**Keywords:** alien vegetation, biochar, eco‐friendly, electrocatalysts, proton exchange membrane fuel cells

## Abstract

Invasive alien vegetation brought about by various human activities has grown to be a significant threat to the ecosystem and its diversity; therefore, control strategies to combat this threat are being explored. This review aims to investigate the prospect of using biochar specifically from alien vegetation as a support material for the proton exchange membrane (PEM) fuel cell electrocatalyst, highlighting the need to move to green energy and invest in Eco conservation. The use of biochar derived from alien vegetation as carbon support for the platinum (Pt) electrocatalyst for PEM fuel cells is an interesting field that is slowly gaining momentum. Biochar has the potential to be used as a carbon support due to its high specific surface, area, and intrinsic property needed for an electrocatalyst support. The current widely used electrocatalyst, which is Pt supported on carbon black, has shown to suffer from corrosion which weakens the bond between the support and the Pt nanoparticles, leading to instability and resistance; therefore, alternative supports are needed also to decrease the Pt loading as it is expensive. The focus of this review is on the benefits and prospects of these cheap green resources in increasing efforts to conserve the environment.

## Graphical abstract

Biochar derived from woody alien vegetation has been used as a support material for proton exchange membrane fuel cells. This study focuses on the prospects of using biochar specifically from alien vegetation as an alternative for carbonaceous support material to convert and store electrochemical energy.

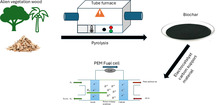

## 1 Introduction

The world is facing a serious problem of continuous increase in energy demands due to the increasing population, which has put enormous pressure on fossil fuel reserves.^[^ ^1^, ^2^ ^]^ Using fossil fuels to meet the growing energy demand has led to a continuous increase in greenhouse gases such as carbon dioxide (CO).^[^ ^2^ ^]^ In May 2024, CO_2_ concentration measured a staggering average of 426.7 ppm, a record exceeding previous records and indicating an urgent need for cleaner energy alternatives.^[^ ^3^, ^4^ ^]^ The environmental consequences of rising CO_2_ levels include global warming, extreme weather conditions, and ecosystem disruptions. Researchers are exploring sustainable energy technologies that minimize carbon emissions to mitigate these effects.^[^ ^3^, ^4^ ^]^

**Figure 1:**
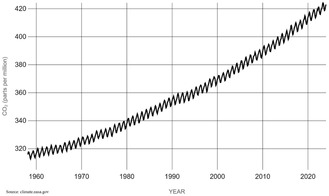
Increasing carbon dioxide concentration in the atmosphere over the years by NASA.^[^ ^5^ ^]^ Data Source: Copyright National Aeronautics and Space Administration available from **https://climate.nasa.gov/vital‐signs/carbon‐dioxide/**.

**Figure** **1** shows the increasing trend in CO_2_ concentration on the earth's surface over the years, as measured by NASA.^[^ ^5^ ^]^

Fuel cells have emerged as a promising alternative energy source due to their high efficiency and near‐zero emissions. Fuel cells are electrochemical cells that provide usable electrical energy from the chemical energy contained in the fuel through a series of redox reactions.^[^ ^6^, ^7^ ^]^ Many different types of fuel cells are differentiated by electrolytes and electrocatalysts. The common types of fuel cells are solid oxide fuel cells, alkaline fuel cells, molten carbonate fuel cells, direct alcohol fuel cells, proton exchange membrane fuel cells (PEMFCs), etc. Among the various types, proton exchange membrane (PEM) fuel cells have gained significant attention for their ability to generate clean energy, particularly when hydrogen is used as fuel. Unlike conventional combustion‐based power generation, PEM fuel cells convert chemical energy directly into electricity through electrochemical reactions, producing only heat and water as byproducts. These attributes make PEM fuel cells a viable solution for reducing carbon footprints in various applications, including transportation and stationary power generation.^[^ ^8^, ^9^ ^]^

A key challenge in fuel cell technology is the development of cost‐effective and sustainable materials for fuel cell components, particularly electrocatalysts and support materials. Biochar, a carbon‐rich material produced from the thermal decomposition of biomass under limited oxygen conditions, has gained attention as a green and economical carbon source for energy applications^[^ ^10^ ^]^ The biochar can be used as a support material in fuel cells and can enhance catalyst stability, conductivity, and overall performance while promoting carbon circularity.^[^ ^11^ ^]^ There have been studies that have investigated the prospect of using biochar as a support material, particularly in fuel cells and batteries^[^ ^11^, ^12^, ^13^ ^]^

In South Africa, alien woody species such as *Eucalyptus*, *Pinus*, and *Acacia* have altered local ecosystems by outcompeting native vegetation and depleting water resources. These species are responsible for ≈16% of the 1.444 million cubic meters of water lost annually due to invasive species.^[^ ^14^ ^]^ Converting this biomass into biochar offers a dual‐benefit strategy because it addresses environmental problems while also offering a renewable carbon source for energy purposes.^[^ ^15^, ^16^ ^]^

This work investigates the green synthesis of biochar made from alien vegetation wood and its use in PEM fuel cells. By exploring the physicochemical features and electrochemical performance of biochar‐based materials, this research seeks to contribute to the development of sustainable and efficient energy solutions while encouraging ecological restoration through invasive species management.^[^ ^17^, ^18^ ^]^

## 2 Biochar Production

Depending on the intended application, Biochar can be derived from a wide range of biomass feedstocks. The production of biochar from feedstock biomass involves many complex steps that depend on the nature of the feedstock biomass, the reaction medium, and the method applied. There have been reviews on biochar production and activation in recent times.^[^ ^19^, ^20^, ^21^, ^22^ ^]^

### 2.1 Thermochemical Biochar Production

Biochar results from the heating of a significant amount of organic biomass, at very high temperatures without much or no oxygen present.^[^ ^23^, ^24^ ^]^ Some thermochemical methods that are used to prepare biochar include hydrothermal carbonization, gasification, torrefaction, and pyrolysis. These procedures, depending on the biomass feedstock material and their conditions, have a direct impact on the composition of the resulting biochar at the end of the process.^[^ ^25^ ^]^ Pyrolysis is the most used green thermochemical method for biochar production from biomass feedstock and is the focus of this review.

#### 2.1.1 Pyrolysis

Biochar is a carbon‐rich matter that is produced through the pyrolysis process in most cases, which involves heating organic matter in the absence of oxygen.^[^ ^23^, ^24^ ^]^ The resulting biochar has many applications, such as soil amendment, catalyst support, and carbon sequestration. It can be further processed to produce bio‐oil. Learning how to produce biochar with desirable characteristics is of considerable interest.^[^ ^25^ ^]^

**Figure 2:**
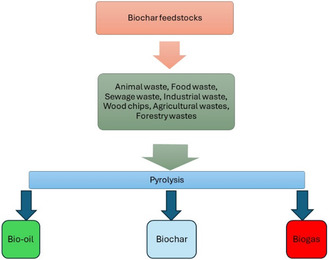
Illustrative diagram showing the possible pyrolysis products of biochar synthetic routes from different biomass feedstocks.

Different organic biochar feedstocks can be used to produce biochar by pyrolysis, as shown in **Figure** **2**. Many variables affect the type of biochar that is formed during pyrolysis, including biomass pretreatment, reactor type and size, pressure, and reactor residence time. Pyrolysis has an advantage over other processes such as hydrothermal carbonization or hydrothermal liquefaction, as it can be used for a variety of biomass and waste materials.^[^ ^25^ ^]^ One of the main benefits of biochar being a green carbon source is its high energy content, which is comparable to that of coal. It has been found to have a higher heating value and its use in combustion or gasification processes can generate heat.^[^ ^25^, ^26^ ^,^ ^27^ ^]^ Biochar also helps reduce greenhouse gas emissions by sequestering carbon in the soil, thus compensating for carbon emissions from biochar combustion, which plays an important role in mitigating climate change.^[^ ^28^, ^29^ ^]^

Pyrolysis also plays a key role in bio‐oil production. It involves heating biochar in the presence of a catalyst and a carrier oil, such as vegetable oil.^[^ ^29^ ^]^ The resulting product is a liquid fuel that can be used in a variety of applications, including as a substitute for diesel fuel in engines. Bioenergy has several advantages over fossil fuels. As it is a renewable resource, it is not subject to the same depletion concerns as fossil fuels. Furthermore, bioenergy can help reduce greenhouse gas emissions, since carbon released during bioenergy combustion is offset by carbon absorbed during plant growth that is used to produce energy. However, biochar can also be a source of carbon, which can be extracted and used for various applications, such as the use of carbon‐based support material in electrocatalysts for fuel cells.^[^ ^30^, ^31^, ^32^ ^]^ Gasification, slow pyrolysis, intermediate pyrolysis, and fast pyrolysis are the techniques mostly preferred as seen in **Table** **1**.^[^ ^33^ ^]^

**Table 1:** Showing various pyrolysis techniques and processes for producing biochar at different temperatures, residence times, and proportions of the product.^[^ ^32^ ^]^

**Process**	**Temperature (** °C)	**Residence time**	**Products** **Liquid (bio‐oil) (%)**	**Products** **Solid (biochar) (%)**	**Products (biogas/syngas) (%)**
Fast pyrolysis	300–1000	Short (<2 [s])	75	12	13
Intermediate pyrolysis	500	Moderate (10–20 [s])	50	25	25
Slow pyrolysis	100–1000	Long (5–30 min)	30	35	35
Gasification	> 800	Moderate (10–20 [s])	5	10	85

The common characteristics of slow pyrolysis often involve a heating temperature range of between 300 and 900 °C with a ramp time range of 5–7 °C ${\text{min}}^{-1}$, and residence times are usually more than 1 h. Slow pyrolysis as compared to fast pyrolysis yields high amounts of biochar and relatively small amounts of oil and syngas (biogas) making slow pyrolysis the preferred method for biochar production. In a study by Aziz et al. they used pinecones from the genus *Pinus* as feedstock to produce modified biochar under slow pyrolysis conditions and showed good antibiotic removal efficiency in water.^[^ ^34^ ^]^

Fast pyrolysis on the other hand consumes less energy due to its high ramp times of ≈200 °C min^−1^ with short residence times. Biochar that results from fast pyrolysis has a high carbon content with good stability even though the biochar yield is quite low as fast pyrolysis favours the formation of bio‐oil; in a study by Heidari et al. they used *Eucalyptus grandis* to study how the conditions of fast pyrolysis affect the bio‐yield.^[^ ^35^ ^]^ Gasification can also be used to produce biochar at very high temperatures, above 800 °C, using gasifying agents as catalysts such as oxygen and steam, resulting in the formation of syngas, a major product relative to biochar.^[^ ^33^, ^36^, ^37^ ^]^

## 3 The Socioeconomic and Ecological Impact of Alien Vegetation and the Benefits of Utilizing it for Biochar Production

Invasive alien vegetation are plants that are brought into an unfamiliar or nonnative habitat and have the potential to proliferate and harm the environment, the economy, or public health. Typically, invasive alien plants exhibit the following traits, that is, high genetic diversity and strong ecological adaptability, fast and robust reproduction, and can reproduce in unfavorable environments.^[^ ^38^ ^]^ Invasive alien plants are highly adaptive and can be found in nonnative environments due to deliberate, inadvertent, or accidental human actions. Because they do not have natural enemies and an ideal habitat, invasive alien plants often have a significant competitive advantage over native plants after successfully establishing a new home. Invasive alien plants can simultaneously create a variety of organic acids, allergenic chemicals, and hormones that can disrupt the relationship between the community of native plants and the ecosystem around them.^[^ ^38^ ^]^ Due to these adverse effects that alien invasive vegetation has on the ecosystem, there have been ways that have been introduced to combat its spread. Invasion of invasive alien plants poses a significant danger to the socioeconomic and ecology of a country 46. An article published by O’Connor and van Wilgen stated that an invasion of South Africa's grasslands by invasive woody alien plants, such as *Acacia*, *Eucalyptus*, and *Prosopis* species, may adversely impact livestock grazing and thereby reduce livestock production by an estimated ZAR 340 million (USD ± 19.2 million) per annum.^[^ ^39^ ^]^ This is a classic example of the adverse effects of these invasive woody alien vegetations. Invasive species in cities can affect ecosystem functions, including water filtering, flood mitigation, and coastal protection. Invasive plants can cause floods by clogging streams and canals, increasing the danger of fire and soil erosion.^[^ ^39^ ^]^

Invasive plant management techniques that are frequently employed include chemical control, which uses chemicals to kill or control invasive plants, mechanical control removal, which uses artificial or mechanical cleaning, and biological control, which introduces additional biological agents to limit the growth of invasive plants.^[^ ^40^ ^]^ Some common uses for invasive plants are composting, feeding, activated carbon, and biochar.^[^ ^41^ ^]^ More affordable and effective techniques to deal with invasive species are desperately needed, as existing methods to do so require a large amount of labor and material resources.^[^ ^42^, ^43^ ^]^

### 3.1 Common Genre of Woody Alien Vegetation in Southern Africa

Several common invasive woody alien vegetations have found homes in the southern parts of the African continent. This is due to the deliberate and unintentional spread of species over their natural dispersion habitats, facilitated by human activities, such as agriculture, recreation, international trade, and transportation.^[^ ^44^ ^]^ Some of the commonly found invasive woody alien vegetation is mostly of the genus *Pinus*, *Eucalyptus*, *Prosopis*, and *Acacia* spp.^[^ ^45^ ^]^ **Table** **2** shows the distribution of some common woody alien vegetation in the southern parts of the African continent.

**Table 2:** Some of the common distribution of some common woody alien vegetation in Southern Africa and their applications studies.

**Genus and species** **of alien vegetation found in Southern Africa**	**Origin**	**Distribution in Africa**	**Some application studies**
*Acacia* *Acacia mearnsii*	Southeastern Australia and Tasmania	Southern Africa^[^ ^82^ ^]^	• Soil remediation^[^ ^46^, ^83^ ^]^
*Prosopis* L. *Prosopis glandulosa* *Prosopis juliflora*	North and Central America	Southern Africa^[^ ^82^, ^84^ ^]^	• Soil remediation^[^ ^47^ ^]^
Eucalyptus spp. *Eucalyptus camaldulensis* *Eucalyptus cladocalyx* *Eucalyptus diversicolor* *Eucalyptus grandis* *Eucalyptus lehmannii* *Eucalyptus paniculata*	South Australia	South Africa, Cape Town South Africa, n.d.^[^ ^85^, ^86^ ^,^ ^87^ ^]^	• $ZnCl_{2}$ modified biochar derived from the *Eucalyptus* tree bark as an adsorbent of Cr (IV) in aqueous solutions^[^ ^47^ ^]^ • *Eucalyptus* biochar activated by KOH for the removal of phenols from waste waters^[^ ^48^ ^]^
*Pinus* L. *Pinus radiata* *Pinus patula* *Pinus taeda* *Pinus illiotti* *Pinus pinaster* *Pinus halepensis* *Pinus pinea*	North America and Europe	Southern Africa^[^ ^81^, ^86^ ^]^	• Use of biochar from pinecones in the removal of antibiotics in water^[^ ^33^ ^]^ • Biochar derived from *Pinus patula* wood as an adsorbent for polluted water by a malachite green dye^[^ ^49^ ^]^

Some physical characterizations of woody alien vegetation biochar; in a study by Pituya et al. they improved the surface area of sandy soil using biochar derived from *Acacia* wood as it had a better specific surface area of 370.37 m^2^ g^−1^ as compared to biochar derived from coconut shells which had a specific surface area of 347.96 m^2^ g^−1^ with pyrolysis conditions of 500 °C for 2 h.^[^ ^45^ ^,^ ^46^ ^]^ In another soil remediation study, Saffeullah et al. used Prosopis wood biochar to improve the mineral content of the soil.^[^ ^47^ ^]^ In a study by Yussuff et al. they derived biochar from a *Eucalyptus* tree bark at a pyrolysis temperature of 500 °C for 1.5 h that was then activated with ZnCl_2_ the biochar had a specific surface area of 217.3 m^2^ g^−1^ and was used as an adsorbent for Cr (VI) from aqueous solutions.^[^ ^48^ ^]^ In another study using *Eucalyptus* wood biochar derived at 550 °C for 2 h and then activated with KOH at 800 °C, it had a specific surface area of 2048 m^2^ g^−1^ that is quite remarkable; the biochar was used to remove phenols from wastewaters.^[^ ^49^ ^]^ The *Pinus patula* wood biochar used to remove the dye in wastewater, and the biochar derived from the *Pinus patula* wood pellets had a specific surface area of 367.33 m^2^ g^−1^ compared to the one extracted from the *Pinus patula* wood chips, which had 233.56 m^2^ g^−1^.^[^ ^50^ ^]^ With surface area being an intrinsic material characteristic in catalysis, this result gives hope that biochar can support PEM fuel cells.

In many African countries, control strategies have been put to try and control the increasing number of these alien species, such as the working for water (WfW) program in South Africa.^[^ ^41^ ^]^ The South African Government's WfW initiative was launched in 1995. The purpose of the program is to monitor the effects of alien invasive vegetation in South Africa on terrestrial and freshwater ecosystems.^[^ ^41^, ^51^ ^]^ In severe situations, these alien plants outcompete our native plant life and eventually cause their extinction. The Department of Environmental Affairs oversees the initiative, which is implemented in the nine provinces and collaborates with other government agencies and commercial companies.^[^ ^51^ ^]^ In addition to creating jobs for the local community, the initiative also helps process the gathered plant material. The South African government's WfW effort lists 198 of the ≈9000 plant species that have been brought to the country as an invasive initiative.^[^ ^41^, ^51^ ^]^

**Figure 3:**
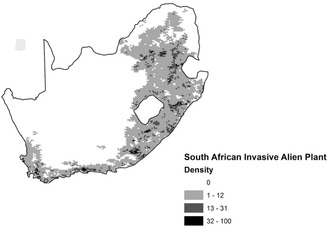
The amount and density (tons/hectares) of woody plants resulting from the invasion of alien plants in South Africa with the province of the Northern Cape excluded.^[^ ^45^ ^]^

The primary woody invasive alien plant species are from the genus *Pinus*, *Eucalyptus*, and *Acacia*, all of which were included in extensive afforestation projects in the past, with the most alien plant invasions occurring in habitats with higher rainfall, while *Prosopis* spp. are the dominant invaders in drier places.^[^ ^45^ ^]^ Figure 2 shows the distribution of alien vegetation in South Africa taken from the 2010 report of the National Invasive Alien Plant Survey (NIAPS) conducted by Kotze et al.^[^ ^52^ ^]^ This report displayed 27 alien plant taxa's condensed‐hectare coverage. To determine the geographical distribution or density of woody matter associated with alien vegetation invaders, aerial surveys were carried out in tertiary basins.^[^ ^52^ ^]^ The Northern Cape was not covered in its entirety by the NIAPS research. In this report, common woody alien vegetation with >20% lignin was prioritized from the genus *Pinus*, *Poplar* spp., *Eucalyptus*, *Hackea*, *Prosopis*, and *Acacia* (see **Figure** **3**), which shows the distribution in South Africa. In this report, it was also found that the mostly distributed Woody Alien Vegetation is from the genus *Eucalyptus* spp., *Pinus* spp. and *Acacia*.

## 4 Applications of Biochar in Energy Production and Storage Systems

### 4.1 Electrochemical Water Splitting

Water splitting is considered the most promising solution for the preparation of hydrogen energy; nevertheless, traditional electrocatalysts are hampered by issues of high overpotential and low efficiency for hydrogen generation. Biochar is used as an electrocatalytic and photocatalytic support to split water to produce hydrogen and oxygen. When a heteroatom is incorporated into the biochar structure, more reaction–active sites are formed for the hydrogen evolution reaction (HER).^[^ ^53^ ^]^

**Figure 4:**
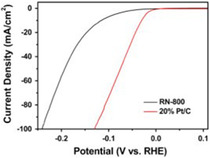
Glassy carbon electrode equipped coated RN‐800 or 20% Pt/C polarization curves for HER in 0.5 m H_2_SO_4_.^[^ ^54^ ^]^

In a study by Zhou et al. they used biochar from peanut root nodule calcined at 800 °C (RN‐800) and was doped with sulfur (S‐doped) and nitrogen (N‐doped) for application as an electrocatalyst for HER.^[^ ^54^ ^]^ Doped biochar showed a good electrochemical surface area of 27.4 mF cm^*−*2^ and an onset potential of −0.027 V (vs. reversible hydrogen electrode (RHE)) and achieved a current density of 10 mA cm^−2^; the overpotential was 116 mV for the HER in 0.5 m H_2_SO_4_ as an electrolyte, which can be compared to the commercial Pt/C catalyst at a Pt loading of 20 wt%^[^ ^54^ ^]^ (see **Figure** **4** showing the HER curves comparing Biochar with the commercial Pt/C).

In a study by Yang et al.^[^ ^53^ ^]^ cobalt oxide nanoparticles were reduced in watermelon peel biochar to create CCW‐x nanocomposites, where x is the temperature of pyrolysis of the watermelon peels. When CW‐700 biochar was used as electrocatalytic material, its overpotential for HER was only 111 mV at 10 mA cm^−2^ which was greater than that of Pt/C currently explored in the literature.

**Figure 5:**
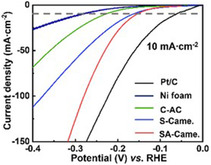
HER LSV curves for 1 m KOH at 10 mA cm^−2^ CAC, S‐Came, and SA‐Came and Pt/C.^[^ ^55^ ^]^

Sulfur self‐doped biochar from *Camellia japonica* (camellia) can be used as a catalyst and can be used for general water‐splitting reactions (SA‐Came). In 1 m KOH, the electrocatalyst HER activities of the electrocatalyst at 10 mA cm^−2^ namely, C‐AC, S‐Came, and SA‐Came, were investigated through linear sweep voltammetry (LSV) (see **Figure** **5**); SA‐Came (red) biochar showed a great over potential of 154 mV which was lower as compared to the other biochar as comparable to the commercial Pt/C at 10 mA cm^−2^.^[^ ^55^ ^]^

In a study by An et al. they studied the biochar from sunflower seed shells together with Mo_2_C nanoparticles. For HER, the electrocatalyst showed great activity with a current density of 10 mA cm^−2^ with a low 60 mV over potential; most notably, this catalyst maintained exceptional durability and almost a faradaic efficiency.^[^ ^56^ ^]^ The most effective water‐splitting catalysts commercially available are still more effective than the biochar catalyst. However, biochar is promising as a readily available and inexpensive substitute catalyst material for the generation of oxygen and hydrogen; therefore, more work needs to be done for biochar catalysts to compete fully with commercial Pt/C catalysts.

### 4.2 Rechargeable Batteries

Because biochar can be produced sustainably and at a lower cost than traditional graphite materials and because of its high specific charge storage capacity, it is a suitable carbon alternative material for a variety of rechargeable batteries.^[^ ^57^ ^]^

**Figure 6:**
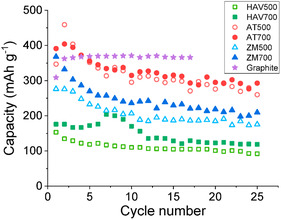
The cycling performance of aquatic vegetation biochar and compared to graphite.^[^ ^59^ ^]^

A study by Zhou has shown that using biochar derived from wheat stalks as an anode material of the lithium–ion battery presents a multitude of lithium–ion storage sites, fast electron and *Li* ^+^ ion transfer, a flatter study of the level voltage, and reduced voltage hysteresis. The *Li* ^+^ the biochar‐based ion batteries described here had a reversible capacity of 502 mAh g^−1^, which outperforms the theoretical capacity of graphite; however, the rate and cycling ability were exceptional.^[^ ^58^ ^]^ In a separate investigation by Belmesov et al. the researchers explored biochar sourced from the ocean plant Ahnfeltia tobuchiensis, specifically pyrolyzed at 700 °C (AT‐700). This biochar demonstrated an impressive reversible capacity of 391 mAh g^−1^, which decreased to approximately 300 mAh g^−1^ after 25 cycles. This performance was comparable to graphite and outperformed other ocean‐derived biochars studied, primarily those from seagrass Zostera (ZM) and Ruppia (HAV), which were pyrolyzed at temperatures of 500 and 700 °C. In **Figure** **6**, the letters represent the type of ocean plant, and the numbers denote the pyrolysis temperature.^[^ ^59^ ^]^

In another work by Pongpanyanate et al. they used sugar cane bagasse biochar, where bagasse biomass‐derived biochar was N‐doped and the MnO_2_ nanoparticles were reduced on the surface of biochar in different concentrations: 5, 10, 40, and 100 mm with KMnO_4_ as a precursor resulting in x–MnO_2_/NBGC nanocomposites with *x* ‐representing the loading concentration of the nanoparticles.^[^ ^60^ ^]^

**Figure 7:**
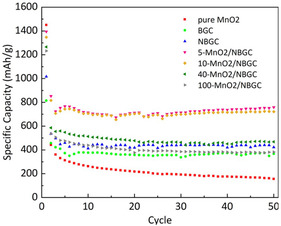
Evaluation of cycling performance of carbon materials derived from bagasse (BGC), nitrogen‐doped carbon materials derived from bagasse (NBGC) at different MnO_2_ nanoparticle loadings, and the pure MnO_2_ nanoparticles that were synthesized, all at a current density of 186 $\mathrm{mA}\;g^{-1}$.^[^ ^60^ ^]^

In the study, it was found that the 5‐MnO_2_/NBGC nanocomposite showed the greatest electrochemical performance with a reversible capacity of 760 mAh g^−1^ at a current density of 186 mA g^−1^ compared to the others (see **Figure** **7**). It showed good reversibility with ≈100% coulombic efficiency and achieved reversible capacities of 488 and 390 mAh g^−1^ in the cycle testing at 372 and 744 mA g^−1^ current densities, respectively, over 150 cycles.^[^ ^60^ ^]^ It is crucial to note that more research is thought to be required to boost the specific capacity by designing high‐voltage cathodes and maybe low‐potential anodes made of biochar.

### 4.3 Supercapacitors

Today's supercapacitors are equipped with fast charging and discharging speeds, great power density, and outstanding durability. Consequently, supercapacitors are widely used in several fields that need a high‐power density, such as electrical vehicles.^[^ ^57^ ^]^ For the fabrication of electrodes in supercapacitors, it is advantageous to use carbon materials with a substantial specific surface area exceeding 2000 m2 g^−1^, such as activated carbon. In such capacitors, a specific capacitance ranging from 250–350 F g^−1^ has been recorded.^[^ ^57^ ^]^ Therefore, the aim is to synthesize inexpensive functional carbons with improved specific capacitance. In this context, recent research has demonstrated the great potential of biochar‐based products to replace traditional activated carbon. Due to the porous structure of biochar and high conductivity, it can be a promising material for high‐specific capacitance supercapacitors.^[^ ^61^ ^]^

**Figure 8:**
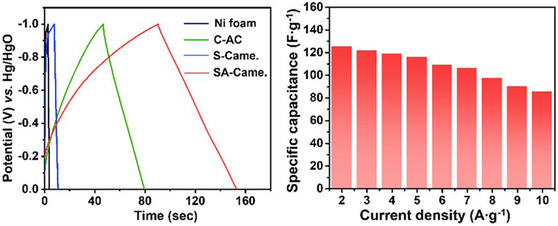
The GCD curves at a current density of 2 A g^−1^ of all the synthesized electrode materials (left) and the plot of the specific capacitance retention of the GCD curves against current density (right).^[^ ^55^ ^]^

Xia et al. conducted research on heteroatom‐doped biochar, using a self‐doped sulfur biochar derived from Camellia japonica (camellia) as an electrode material for supercapacitors, which they designated as SA‐Came. Among the various electrode materials evaluated, it demonstrated a specific capacitance of 125.42 F g^−1^ at a current density of 2  A g^−1^ as evidenced by the Galvanostatic charge‐discharge (GCD) test, see **Figure** **8**. It showed greater cyclic stability in a three‐electrode system using 1 m KOH as an electrolyte. It also showed a high energy density of 34.54 Wh kg^−1^ at a power density of 1600 W kg^−1^ as a symmetric hybrid supercapacitor device at a potential window of 0–1.6 V.^[^ ^55^ ^]^

In another study by Raj et al.^[^ ^62^ ^]^ they investigated the application of biochar to supercapacitors by exploring biochar derived from *Prosopis juliflora* species. This biochar was activated and doped with Thiourea, leading to an increase in heteroatoms mainly sulfur and nitrogen in the structure at 600 °C of the biochar with a surface area of 570 m^2^ g^−1^. From the GCD studies, the electrode material had a specific capacitance of 246 F g^−1^ at 1 A g^−1^. There was an improved retention rate of 96.74% for 3000 cycles. Biswal et al.^[^ ^63^ ^]^ They studied biochar derived from dead leaves of the Azadirachta indica plant. Biochar showed great results, as it had a remarkably high specific capacitance of 400 F g^−1^ and an energy density of 55 Wh k*g* ^−1^ at 0.5 A g^−1^ current density using aqueous 1 m H_2_SO_4_ as an electrolyte.^[^ ^63^ ^]^ Despite these encouraging outcomes, research is conducted to understand the structure‐property link and further increase the capacitance. This might be accomplished by synthesizing a geometric composite with other materials.

### 4.4 Fuel Cells

PEMFCs are a rapidly developing next‐generation energy conversion technology with many potential uses, particularly in electric cars. Toyota and other leading manufacturers have been at the forefront of the use of hydrogen‐powered automobiles.^[^ ^64^, ^65^ ^]^ The high efficiency of energy conversion, superior performance at low operating temperatures below 80 °C, absence of noise and pollution, among other advantages, have heightened the research interest in PEMFCs.^[^ ^7^ ^]^

The main parts of a PEMFC that make up the cell are the anode and the cathode with electrolyte media all connected by an external circuit. With the parts that make up PEMFCs, there is a membrane electrode assembly that is the central component of PEMFCs, made up of the catalyst layer, the gas diffusion layer, and the electrolyte membrane (PEM).^[^ ^65^, ^66^, ^67^ ^]^

**Figure 9:**
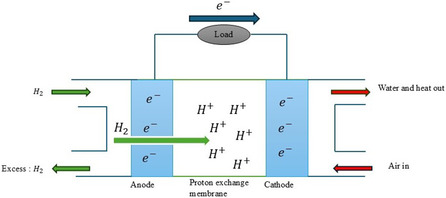
The overview operation of a typical PEMFCs.

The overview operation of a typical PEMFC (see **Figure** **9**), occurs when gaseous hydrogen is supplied to the anode (see Equation (1)), where it is adsorbed to the catalyst's surface. Each hydrogen atom that has been adsorbed loses an electron (*e* ^*−*^) and emerges from the metal surface as a proton (*H* ^+^). Protons flow through the PEM to the cathode, whereas electrons go to the cathode as current via the external circuit. Oxygen is adsorbed onto the catalyst surface when air is supplied to the cathode (see Equation (2)). After this bound oxygen is reduced by incoming electrons and protonated by entering *H* ^+^, water is made and freed from the catalyst surface.^[^ ^6^, ^7^, ^64^, ^65^ ^]^ Because the medium around the fuel cell is hydrophobic, this water is compelled to leave the fuel cell as a byproduct.Anode:

(1) $$H_{2}\to 2H^{+}+2e^{-}$$

Cathode:

(2) $$\frac{1}{2}O_{2}+2e^{-}+2H^{+}\to H_{2}O$$

Overall:

(3) $$H_{2}+\frac{1}{2}O_{2}\to H_{2}O$$

The main reaction mechanisms behind the functioning of the PEMFCs are the oxygen reduction reaction (ORR) occurring at the cathode and the hydrogen oxidation reaction (HOR) occurring at the anode; both reaction mechanisms utilize platinum as a catalyst (see Equation (3)) showing the overall reaction. Pt often exists as nanoparticles on the surface of slightly larger carbon materials that serve as supports for a platinum‐carbon‐supported electrocatalyst.^[^ ^7^ ^]^

The HOR occurs when the hydrogen gas enters the fuel cell and travels to the Pt anode, where the HOR occurs. In this instance, the hydrogen–hydrogen bond breaks as it adsorbs onto the Pt electrode, providing adsorbed atomic hydrogen on the surface of the catalyst (*H* ^*^) (see Equation (4)), with (*) representing the adsorption site or the adsorbed hydrogen occurs.^[^ ^7^ ^]^

(4) $$\frac{1}{2}H_{2}+*\to H^{*}$$

The removal of electrons from the adsorbed hydrogen species H* results in their protonation, causing the hydrogen atoms to leave the catalyst surface in the form of *H* ^+^ ions (refer to Equation (5)).

(5) $$H^{*}\to H^{+}+*+e^{-}$$

Therefore, the reaction kinetics of the HOR on a Pt electrode in PEMFCs is extremely quick. Even at extremely low Pt loadings of less than 5 mV loss @ Pt anode loadings of less than 0.05 mg cm^−2^, voltage losses are vanishingly tiny. Therefore, the cathode process ORR has long been the primary focus of researchers.^[^ ^7^, ^65^ ^]^

Fuel cells have sluggish cathodic ORR kinetics. To ramp up the reaction kinetics, platinum‐based catalysts such as carbon‐supported platinum (Pt)‐based catalysts must be used. The current problems with Pt‐based catalysts are high costs and short durability; therefore, they are emerging as key factors impeding the effective commercialization of fuel cells.^[^ ^7^, ^65^ ^]^

The two most prevalent ORR mechanism processes are a four‐electron reduction process that produces water directly from oxygen and a two‐electron reduction process that converts oxygen to hydrogen peroxide and then water.^[^ ^7^, ^68^, ^69^, ^70^ ^]^

Intermediate products produced by the two‐electron reduction process, such as hydrogen peroxide, oxidize the electrode and membrane materials and harm the catalyst structure in addition to lowering the reaction efficiency. Consequently, this decreases the catalytic efficiency of the fuel cell and shortens its useful life.^[^ ^65^ ^]^ The (*) represents the adsorption site on the catalyst surface.

The four‐electron process is as follows.

(6) $$O_{2}+4e^{-}+4H^{+}\to 2H_{2}O$$

(7) $$O_{2}+4e^{-}+4H^{+}\to OOH^{*}+3H^{+}+3e^{-}$$

(8) $$\to O^{*}+2H^{+}+2e^{-}\to OH^{*}+H^{+}+e^{-}\to 2H_{2}O$$

The two‐electron process is as follows.

(9) $$O_{2}+2e^{-}+2H^{+}\to 2H_{2}O_{2}$$

(10) $$O_{2}+2e^{-}+2H^{+}\to OOH^{*}+H^{+}+e^{-}\to H_{2}O_{2}$$

The four‐electron process does not lead to fuel cell corrosion since it creates water as a product. Because it is irreversible and has a faster rate of energy conversion, it is often regarded as the preferable reaction route. For this reason, a crucial need to guarantee the effectiveness of Pt/C catalysts is the prevalence of the four‐electron reaction.^[^ ^69^, ^70^ ^]^

The four‐electron process is still the generally acknowledged reaction mechanism for Pt‐based catalysts, although the two‐electron reaction mechanism has been discovered for Au catalysts with low activity. The four‐electron mechanism is the optimal ORR reaction pathway. PEMFCs make higher working potentials and current efficiency possible due to the lack of peroxide formation, which also helps to prevent the deterioration of electrodes and electrolyte films.^[^ ^71^ ^]^

To a certain extent, the stability, durability, and activity of Pt/C catalysts that are readily available for fuel cell catalysis can be ensured, with Pt supported on carbon black. The catalyst consists of platinum nanoparticles loaded onto a carbon support material that should be resistant to corrosion in different media.^[^ ^72^ ^]^ The main obstacles preventing Pt/C catalysts from being widely used are the scarcity of Pt and the high cost.^[^ ^65^ ^]^ During fuel cell operation, Pt metal particles are susceptible to disintegration, growth, agglomeration, and migration, reducing the active surface area and ultimately causing Pt nanoparticles to detach from the carbon support.^[^ ^73^, ^74^ ^]^ Electrochemical Ostwald ripening results in a difference in chemical potentials between nanoparticles of different sizes, leading to the loss‐specific surface. As a result, smaller particles tend to break down in the electrolyte and deposit on bigger particles, increasing the size of the particles.^[^ ^75^, ^76^ ^]^

A portion of the CO found in the hydrogen gas produced by industrial reforming diffuses and adsorbs on the cathode after entering through the anode.Some CO present in the hydrogen gas generated through industrial reforming processes enters via the anode, then diffuses and adheres onto the cathode. This CO contaminates the Pt/C catalyst by blocking its active sites, which in turn decreases the cell's energy conversion efficiency.^[^ ^77^, ^78^ ^]^

The hostile electrolyte environment exposes carbon support materials to oxidation and corrosion. Fuel cells operate under conditions of intense acidity pH less than 1 and high voltage (0.6–1.0 V).^[^ ^65^ ^]^ Biochar offers an alternative as it can be used as a cheaper alternative; biochar is worth exploring to try and solve some of the issues faced by platinum/crystal catalysts.

In research conducted by Hao et al. biochar derived from the *Spartina alterniflora* plant, which is non‐native to China, was used as a carbon source. This biochar was doped with nitrogen and termed Spartina alterniflora N‐doped carbon (SANC) for application in the oxygen reduction reaction (ORR). The material demonstrated impressive electrochemical performance, featuring a half‐wave potential of 0.716 V against RHE with a four‐electron transfer process producing 2.05% H_2_O_2_. The number of electrons transferred (n) during the ORR was 3.96. Furthermore, SANC exhibited excellent stability for ORR, retaining 72.7% of its initial current density after testing for 3000 seconds at 0.7 V versus RHE.^[^ ^79^ ^]^ In another study by Zhang et al. in efforts to develop a sustainable cost‐effective approach, they prepared N‐doped porous biochar as a carbon source derived from mulberry leaves as support for Fe_2_ O_3_ nanoparticles activated at 850 °C for ORR electrocatalysis. In their study, they discovered that the doped biochar performed remarkably for ORR with an onset potential of 0.936 V and a reduction peak potential of 0.776 V versus RHE.^[^ ^80^ ^]^ There has been work done by researchers to establish how will biochar perform as electrocatalyst support for methanol oxidation reaction (MOR); Matthews et al. developed activated biochar from orange peels decorated with Co_3_O_4_ nanoparticles and used it as a support material to a PtRu alloy to form a PtRu/Co_3_O_4_^−^, activated carbon nano‐electrocatalyst, and evaluated its electrochemical performance toward MOR.^[^ ^81^ ^]^ They found that the electrocatalyst displays better electrochemical performance relative to the commercially available catalyst Pt/C. The catalyst had a high current density of 6709 mA mg^−1^ with an onset potential of 0.3 V versus RHE; it also showed good tolerance to CO poisoning with *I*_f_/*I*_r_ ratio of 3.0 the highest and a current retention of 35.1% after 10 000 s of testing.

## 5 Challenges and Future Perspectives

Carbon support materials, such as carbon black, have frequently been utilized as carbon support for electrocatalysts for fuel cells. However, these support materials do have several drawbacks, such as being reduced due to corrosion catalyst performance, which lowers fuel cell durability and overall performance. Among the disadvantages are micropores, which restrict surface accessibility, reduce surface area, and facilitate corrosion at high potentials. To improve the function of the PEM fuel cell, an upgraded or modified carbon material is needed. Biochar's unique properties, namely its high conductivity and vast surface area, make it a viable replacement for the carbon support material that was previously used.

It goes without saying that the efficiency of present biochar catalysts is significantly lower than that of the best water‐splitting catalysts. Nevertheless, it may be employed as an abundant substitute catalyst material to produce oxygen and hydrogen. The electrocatalytic activity of biochar is still rather poor. Gaining a comprehensive grasp of surface chemistry and molecular interactions is necessary for both theoretical and experimental studies to enhance performance. Accurate theoretical models are frequently missing because of the very amorphous nature of these materials and their unknown atomic surface structure. We believe that more study in this area will lead to new scientific perspectives and opportunities, as well as useful applications in many other sectors.

## 6 Conclusion

Renewable energy is one of the solutions that can help meet future energy needs. For renewable energy sources to be used more in society, they must be made cheaper and efficient. Biochar has also been explored as an electrode material and as a carbonaceous support for electrocatalysts, which has been used in certain reactions occurring in biodiesel and biohydrogen production. It is necessary to analyze the synthesis of biochar with specific characteristics. This train of thought about research on biochar‐based material in the renewable energy sector signifies that biochar has a chance of becoming a future energy option. The alien woody biochar has the potential to be a good alternative for electrocatalyst carbonaceous support material for conversion and storage for electrochemical energy. However, this electrocatalyst still needs further research to improve it before it can be used for commercial purposes fully. According to recent studies, invasive alien vegetation and its byproducts can be turned into value‐added products such as biochar, furniture, bioenergy, plant fertilizers, medicinal extracts, etc., as a control strategy to combat their spread.

## Conflict of Interest

The authors declare no conflict of interest.
